# Changes of Brain Structures and Psychological Characteristics in Predatory, Affective Violent and Nonviolent Offenders

**DOI:** 10.3390/tomography8030121

**Published:** 2022-06-08

**Authors:** Ming-Chung Chou, Tien-Cheng Cheng, Pinchen Yang, Rueih-Chin Lin, Ming-Ting Wu

**Affiliations:** 1 Department of Medical Imaging and Radiological Sciences, Kaohsiung Medical University, Kaohsiung 80780, Taiwan; mcchou@kmu.edu.tw; 2 Department of Medical Research, Kaohsiung Medical University Hospital, Kaohsiung 80708, Taiwan; 3 Center for Big Data Research, Kaohsiung Medical University, Kaohsiung 80708, Taiwan; 4 Department of Planning, Ministry of Justice, Taipei 10048, Taiwan; victor@mail.moj.gov.tw; 5 Department of Criminal Investigation, Taiwan Police College, Taipei 11696, Taiwan; 6 Department of Psychiatry, College of Medicine, Kaohsiung Medical University, Kaohsiung 80708, Taiwan; pinchen@kmu.edu.tw; 7 Department of Psychiatry, Kaohsiung Medical University Hospital, Kaohsiung 80708, Taiwan; 8 Master Program in Crime Prevention, Providence University, Taichung 43301, Taiwan; rclin2004@yahoo.com.tw; 9 Department of Radiology, Kaohsiung Veterans General Hospital, Kaohsiung 81362, Taiwan; 10 Faculty of Medicine, School of Medicine, National Yang Ming Chiao Tung University, Taipei 11221, Taiwan; 11 Institute of Clinical Medicine, National Yang Ming Chiao Tung University, Taipei 11221, Taiwan

**Keywords:** gray matter, white matter, violent crime, rectus gyrus, middle frontal gyrus

## Abstract

Purpose: Violent subjects were demonstrated to exhibit abnormal brain structures; however, the brain changes may be different between criminals committing affective (VA), predatory violence (VP), and non-violence (NV). Therefore, the purpose of this study was to compare the differences in brain structures and psychological characteristics between VA, VP, and NV offenders. Methods: Twenty male criminal subjects (7 VP; 6 VA; and 7 NV) offenders; and twenty age-matched male healthy non-criminals were enrolled in this study. All subjects received psychological assessments as well as magnetic resonance imaging scans of the brain. Analysis of variance (ANOVA) was performed to understand the differences among four groups with Bonferroni correction. The voxel-based morphometry and voxel-wise diffusion tensor imaging analyses were performed to compare the gray matter (GM) volume and white matter (WM) integrity between the groups. In significant regions, a Spearman correlation analysis was performed to understand the relationship between the brain changes and psychological scores. Results: The ANOVA analysis showed that AUDIT scores were significantly different among four groups, but no significant group difference was noted after Bonferroni correction. The imaging comparisons further demonstrated that the VP and NV offenders exhibited significant alterations of WM and GM tissues in the rectus and superior temporal gyrus, respectively. In addition, the VP offenders exhibited greater GM volumes than VA offenders in the right middle frontal gyrus, and NV offenders had greater GM volumes than VP offenders in the bilateral thalamus. Conclusion: We concluded that the VA, VP, and NV groups exhibited different degrees of alterations in GM and WM tissues in regions involved in emotion and cognition.

## 1. Introduction

Violent individuals have been demonstrated to exhibit abnormalities in brain structure and function [1,2,3,4]. There have been many studies that showed structural and functional abnormalities in the brains of antisocial individuals. Some hypotheses have proposed a link between antisocial behavior and deficits in certain brain regions, such as the prefrontal cortex, temporal cortex, insula, amygdala, hippocampus, para-hippocampus, anterior cingulate gyrus, and posterior cingulate gyrus [5,6,7]. Among these brain regions, the prefrontal cortex has been recognized as the most crucial brain structure that determines violent and antisocial behaviors in these aggressive individuals [8,9].

The results of a previous meta-analysis showed significantly reduced prefrontal structure and function in antisocial individuals [1]. These findings confirmed the replicability of prefrontal structural and functional impairments in antisocial populations and highlighted the involvement of the orbitofrontal, dorsolateral frontal, and anterior cingulate cortices in antisocial behaviors. Nevertheless, in the meta-analysis study, five studies were performed on antisocial subjects who had conducted nonviolent crimes, and seventeen were performed on antisocial subjects with comorbid psychiatric diagnoses [1]. Therefore, it will be of value to investigate further regarding different violent behaviors and their association with brain abnormalities.

To the best of our knowledge, there has been only one previous brain imaging study that used positron emission tomography to investigate the brain differences between affective, impulsive, and planned violence [10]. The results supported the hypothesis that emotional, unplanned impulsive murderers were less able to regulate and control aggressive impulses generated from subcortical structures due to deficient prefrontal regulation. However, it remains unclear whether the gray matter (GM) volume and white matter (WM) integrity are different among offenders committing crimes with impulsive/affective violence (VA), predatory violence (VP), and non-violent (NV) offenders.

In the present study, we hypothesized that VA and VP offenders may exhibit different brain structures in regions involved in emotion and cognition. Therefore, the purpose of this study was to investigate the differences of GM and WM tissues between VA, VP, NV, and healthy control (HC) groups using voxel-based morphometry (VBM) and voxel-wise diffusion tensor imaging (DTI), respectively.

## 2. Materials and Methods

This prospective study was approved by the local institutional review board of Kaohsiung Veterans General Hospital (protocol number: VGHKS93-CT2-09). This study enrolled 20 right-handed male offenders who were further separated into 3 sub-groups by their records of court verdict: six offenders had committed affective violence (VA group), seven offenders had committed predatory violence (VP group), and seven had committed non-violent crime (NV group). The VA offenders were defined as the subjects who impulsively or affectively committed violent crime. The VP offenders were defined as the subjects who purposely planned to commit violent crime with detailed documentation in the court records. The NV offenders were defined as the subjects who committed non-violent crime based on the documents in court record. In addition, 20 age-matched right-handed male non-criminal healthy controls (HC group) were enrolled for comparisons. All subjects underwent psychological assessments as well as magnetic resonance imaging (MRI) scans of the brain after providing their written informed consent. Individuals with metal implants, claustrophobia, neurological disorders, or any space-occupying lesion in the brain MRI were excluded from this study.

### 2.1. Psychological Assessments

Three psychological questionnaires were arranged for each participating subject to complete. Alcohol consumption was evaluated using the alcohol use disorders identification test (AUDIT) which is a ten-item scale consisting of three dimensions: alcohol consumption, alcohol dependence, and alcohol-related problems. The highest score that can be obtained from AUDIT is 40 [11] and scores above 8 indicate a high risk of alcohol use disorder. The questionnaire correlates highly with other alcohol screening tools and a high internal consistency (0.75 to 0.94) has been reported in various studies [12]. Impulsiveness was assessed using self-report Dickman’s impulsivity inventory (DII) [13]. This instrument is a self-report questionnaire developed to measure two types of impulsivity, namely, functional and dysfunctional impulsivity. Dysfunctional impulsivity is the tendency to make quick decisions and act with less forethought when this tendency is non-optimal or a source of difficulty. Functional impulsivity is the tendency to make quick decisions and act with little forethought when it is optimal and beneficial. It consists of 23 items with a true/false answer and the Cronbach’s alpha was 0.81 for dysfunctional impulsivity, and 0.78 for functional impulsivity. We used the dysfunctional impulsivity score in the final analysis. Hostility was measured using the Buss–Durkee hostility inventory (BDHI), which was an instrument to measure the aggression potential of individuals [14]. It is a 34-point Likert-type self-report inventory with each item scored between 1 and 5. The total aggression level is calculated with the total score and high scores indicate that the aggression tendency is high. We used the total aggression score in our final analysis.

### 2.2. MRI Acquisition

MRI data were acquired from all participants on a 1.5T MR scanner (Signa HDx, GE Healthcare, Milwaukee, WI, USA) with an 8-channel phased-array head coil. Each subject was scanned in a supine position with head-first orientation. In this study, tri-planar scans were firstly performed for localization, then a calibration dataset was acquired for the reconstruction of parallel imaging. After conventional T1-weighted, T2-weighted, and fluid-attenuated inversion recovery images were acquired, whole-brain high-resolution T1-weighted imaging data (TR/TE/TI = 9.1/4.2/500 ms, flip angle = 20°, array coil spatial sensitivity encoding technique factor = 2, field-of-view = 250 × 188 mm, matrix size = 256 × 192, slice thickness = 1.2 mm, number of slice = 124, and number of excitation = 1), and DTI data (TR/TE = 10,000/77 ms, field-of-view = 280 × 280 mm, matrix size = 128 × 128, slice thickness = 4.4 mm, number of direction = 15, number of excitation = 3, acceleration factor = 2, scan time = 8 min) were acquired using 3D magnetization-prepared rapid gradient echo and spin-echo echo-planar diffusion-weighted pulse sequences, respectively.

### 2.3. Image Processing

The high-resolution T1-weighted imaging and DTI data were transferred to a standalone workstation for imaging statistics. In VBM analysis, T1-weighted images were analyzed using the CAT12 (Computational Analysis Tool version 12, http://www.neuro.uni-jena.de/cat/, accessed on 5 June 2022) toolbox. The preprocessing steps of the analysis were field bias modulation, tissue segmentation, diffeomorphic anatomical registration through exponentiated lie algebra-based spatial normalization [15], and spatial smoothing. In this study, default parameters and the East Asian brain template were used in the image preprocessing. Afterwards, the normalized and segmented GM images were statistically compared between the groups on a voxel-by-voxel basis.

In voxel-wise DTI analysis, the image data were firstly corrected for the motion and eddy-current distortion using rigid-body and affine registrations, respectively, run in FSL (FMRIB Software Library, Oxford, UK). Second, the brain parenchyma was extracted using a brain extraction tool based on b0 images. Third, DTI data were then analyzed to obtain fractional anisotropy (FA), axial (AD), radial (RD), and mean diffusivity (MD) using the DTIFIT (FSL, FMRIB, Oxford, UK) tool. FA is an indicator of tissue integrity. AD and RD are the diffusivity in directions parallel and perpendicular to the axons, respectively. MD is the averaged diffusivity of a tensor. Fourth, the FA maps were spatially normalized to a standard coordinate defined by an international consortium for brain mapping-FA template. The normalization was carried out using both linear affine and non-linear demon image registrations, and the corresponding AD, RD, and MD maps were spatially normalized. Finally, the voxel-wise analysis was carried out using the SPM12 toolbox (Statistical Parametric Mapping version 12, https://www.fil.ion.ucl.ac.uk/spm/software/spm12/, accessed on 5 June 2022).

### 2.4. Statistical Analysis

For psychological assessments, one-way analysis of variance (ANOVA) was performed to determine whether there was a significant difference between two groups. The differences were considered significant if corrected *p* < 0.05 with Bonferroni correction. For both VBM and DTI analyses, a voxel-wise two-sample *t*-test was performed to understand the difference of GM volumes and DTI indices between the groups with age as a covariate, and the differences were statistically significant if uncorrected *p* < 0.001 and cluster > 100 voxels. Moreover, in significant regions, a Pearson’s correlation analysis was performed to understand the relationship between the structural changes and the scores in each group. The correlation was considered significant if *p* < 0.05.

## 3. Results

One-way ANOVA analysis revealed that, in psychological assessments, only the AUDIT scores were significantly different among the groups, but no significant AUDIT differences were noted between two groups after Bonferroni correction, as shown in Table 1. Besides, the HC group had significantly higher education levels than other subgroups.

**Figure 1:**
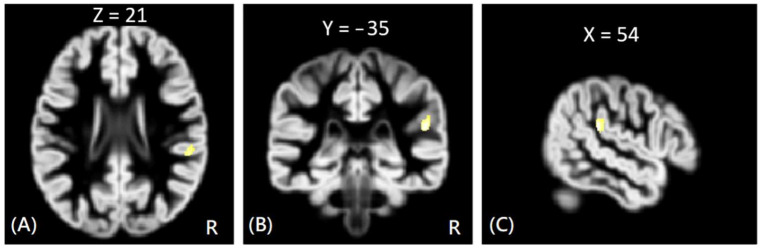
The VBM analysis of GM volume between the NV and HC groups. The yellow-white color indicates the significantly increased GM volumes in the NV group HC in the right superior temporal gyrus. The images are shown in axial (**A**), coronal (**B**), and sagittal views (**C**).

**Figure 2:**
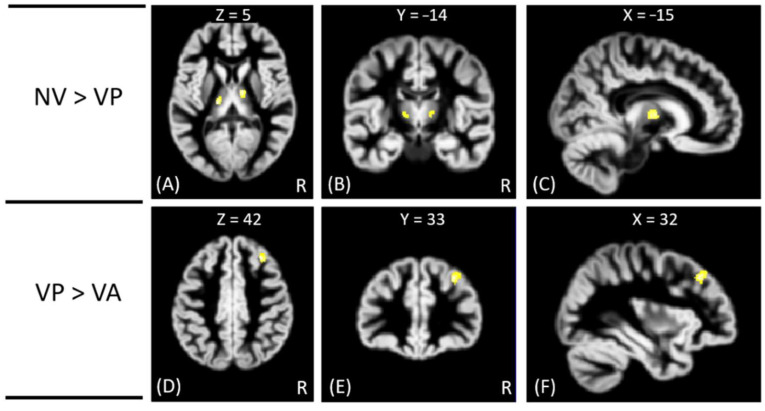
The VBM analysis of GM volume between the VP, VA, and NV groups. The yellow-white color indicates the significantly different GM volumes between VP and NV in the bilateral thalamus (**A**–**C**), and between VA and VP in the right middle frontal gyrus (**D**–**F**). The images are shown in axial (**A**,**D**), coronal (**B**,**E**), and sagittal views (**C**,**F**).

In VBM analysis, the results showed that the offenders (VA, VP, and NV) had significantly greater GM volumes than the HC group in the right superior temporal gyrus. The sub-group comparisons further revealed that only the NV group had significantly greater GM volumes than the HC group in the right superior temporal gyrus, but the VA and VP groups did not exhibit significant change in GM volume as compared to the HC group, as shown in Figure 1. In addition, the NV group had significantly greater GM volumes than the VP group in bilateral thalamus, whereas the VP group had significantly greater GM volumes than the VA group in the right middle frontal gyrus. However, no significant differences in GM volume were noted between VA and NV groups, as shown in Figure 2. The Montreal Neurological Institute (MNI) coordinates of regions with significantly different GM volumes between VA, VP, NV, and HC groups are listed in Table 2. No significant correlation was noted between GM volume and psychological scores in significant regions.

**Figure 3:**
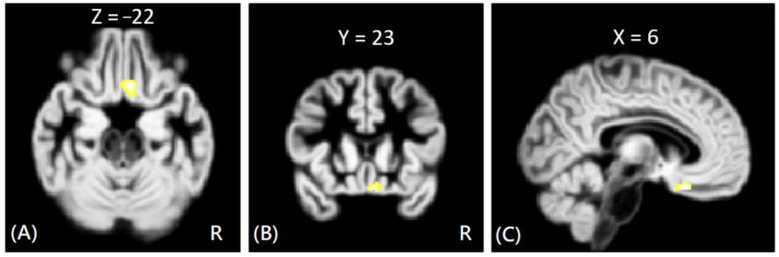
The voxel-wise DTI analysis of AD values between VP and HC groups. The yellow-white color indicates significantly increased AD values in the VP group in the right rectus gyrus. The images are shown in axial (**A**), coronal (**B**), and sagittal views (**C**).

In DTI analysis, the results showed that the offenders (VA + NV + NV) had significantly increased AD, RD, and MD values than those of HC group. The sub-group comparisons further revealed that only the VP group exhibited significantly increased AD, RD, and MD values than the HC group in the right rectus gyrus, as shown in Figure 3. The MNI coordinates of regions with significantly different AD, RD, and MD values between VP and HC groups are listed in Table 3. However, no significant correlation was noted between DTI indices and psychological scores in the significant region.

## 4. Discussion

To the best of our knowledge, this is the first study to investigate changes in brain GM, WM, and psychological characteristics in affective, predatory, and non-violent offenders. Different from previous studies [16,17,18,19,20,21], the present study demonstrated that, although non-violent offenders had slightly higher AUDIT scores than other groups, the psychological characteristics (AUDIT, DII, and BDHI scores) were not significantly different between two sub-groups after Bonferroni correction. In brain structures, the non-violent offenders had significantly increased GM volumes in the right superior temporal gyrus, and the predatory offenders had significantly increased diffusivity in the right rectus gyrus as compared to healthy subjects. Moreover, the non-violent offenders exhibited greater GM volumes than the predatory offenders in the bilateral thalamus, and the predatory offenders had greater GM volumes than affective offenders in the right middle frontal gyrus. These findings highlighted the different involvement of cerebral GM and WM tissues between the three groups.

In the predatory offenders, the DTI results demonstrated that the predatory offenders had significantly higher AD, RD, and MD values than healthy subjects in the right rectus gyrus. The rectus gyrus was previously shown to be associated with depression [22], aggression (irritability and hostility) [23], and attention-deficit/hyperactivity disorders [24]. It is known that the increased diffusivity may indicate that the extra-cellular space was increased likely due to partial axonal loss or vasogenic edema. Therefore, the predatory offenders may have altered right rectus gyrus function due to axonal degeneration, thus indicating possible impact on depression, aggression, attention, and hyperactivity behaviors. Further investigation will be needed to show the relationship between these behavioral characteristics and brain structural changes in violent subjects. In the non-violent offenders, although the VBM analysis demonstrated that all of the offenders (VA, VP, and NV) exhibited increased GM volume in the right superior temporal gyrus than healthy subjects, the sub-group comparison revealed that only non-violent offenders had significantly greater GM volumes than healthy subjects in the gyrus. The superior temporal gyrus was shown to be involved in the perception of emotions in facial stimuli, language, auditory processing, and social cognition processes [25,26]. Therefore, the finding of increased GM volume in the right superior temporal gyrus suggests that the non-violent offenders may exhibit altered functions involved in facial stimuli, language, auditory processing, and social cognition. 

In addition, the affective offenders exhibited a significantly smaller GM volume than the predatory offenders in the right middle frontal gyrus, suggesting that the affective offenders exhibited functions of emotion, attention, and memory different from those in the predatory offenders. The non-violent offenders had greater GM volumes than predatory offenders in the bilateral thalamus. It is known that the thalamus plays an important role in sensory, motor, attention, cognition, and memory functions. The findings may indicate that the predatory offenders exhibited more deteriorations in sensory, motor, cognition, and memory functions than those of the non-violent offenders. 

Moreover, some previous studies demonstrated increased GM volumes in both cortical and subcortical regions [18,19], but others showed reduced GM volumes [16,17,18,19,20]. One previous study further reported no significant changes in violent offenders as compared to healthy subjects [21]. Differently, the present study demonstrated that the non-violent offenders exhibited increased GM volumes, and the affective and predatory offenders did not have significant alterations of GM volume as compared to healthy subjects. The inconsistent GM volume changes between the previous and present studies were likely attributable to the characteristics of enrolled violent subjects, as well as grouping strategy. Specifically, the smaller GM volume of the right middle frontal gyrus in the affective offenders than the predatory offenders further supports the hypothesis that emotional, unplanned impulsive murderers were less able to regulate and control aggressive impulses due to deficient prefrontal regulation, whereas predatory offenders have more prefrontal capacity to regulate and control these impulses [2,10]. Our preliminary findings may shed a light on the understanding of brain structural differences between the affective and predatory offenders. 

There are some limitations to the present study that warrant discussion. First, a small sample size may lead to a low statistical power. A study enrolling more violent subjects will be needed to provide more comprehensive results. Second, this study only enrolled male subjects. Therefore, these results do not reflect brain changes in female subjects. Third, the present study did not evaluate cognitive functions of the enrolled subjects, so the difference of cognitive functions between the violent subjects could not be confirmed. Fourth, the education levels of healthy subjects were higher than the offenders, so the results may have been affected by the education difference between the offenders and healthy subjects. Finally, in DTI analysis, the through-plane resolution (slice thickness) was relatively lower than the in-plane resolution; hence, the results of DTI analysis may be affected by the partial volume averaging in the through-plane direction. In addition, the DTI was acquired with echo-planar imaging pulse sequence, and likely suffered from susceptibility distortions. Thus, the results of the present study might be influenced by the susceptibility distortions.

## 5. Conclusions

This study performed both VBM and DTI analyses to understand the changes in GM volume and WM diffusion, and psychological characteristics in violent offenders. Our results demonstrated that the predatory and non-violent offenders exhibited significant changes in WM diffusion and GM volume in the rectus and superior temporal gyrus, respectively. Moreover, the predatory offenders had greater GM volumes than affective offenders in the right middle frontal gyrus, and that the non-violent offenders exhibited greater GM volumes than predatory offenders in the bilateral thalamus. Therefore, we concluded that the affective, predatory, and non-violent offenders exhibited different patterns of alterations in GM and WM tissues in regions involved in emotion and cognition.

## Figures and Tables

**Table 1:** The demographic characteristics of enrolled subjects.

Group	Age (Years)	Education (Years) *	AUDIT *	DII	BDHI
VA	34.7 ± 12.1	9.4 ± 1.1 ^#^	4.4 ± 5.6	9.4 ± 3.8	14.6 ± 11.1
VP	36.4 ± 8.9	9.4 ± 2.1 ^η^	3.3 ± 1.7	9.3 ± 3.4	16.4 ± 8.3
NV	38.1 ± 8.0	9.4 ± 2.5 ^θ^	10.9 ± 11.3	10.1 ± 4.3	18.9 ± 7.8
HC	34.8 ± 9.6	13.4 ± 2.9 ^#,η,θ^	2.4 ± 2.2	9.5 ± 3.1	19.3 ± 8.7

AUDIT: Alcohol Use Disorders Identification Test. DII: Dickman’s Impulsivity Inventory. BDHI: Buss–Durkee Hostility Inventory. VA: Affective Violence. VP: Predatory Violence. NV: Non-violence. HC: Healthy Controls. Symbols (*^,#,η,θ^) indicate significant difference in ANOVA analysis.

**Table 2:** The Montreal Neurological Institute (MNI) coordinates of regions with significant difference of GM volume between two groups.

	Region	MNI Coordinate			Z Score of Peak-Level Difference
		X	Y	Z	
NV > HC	Rt. Superior Temporal Gyrus	54	−35	21	3.81
NV > VP	Lt. Thalamus	−15	−20	5	3.82
	Rt. Thalamus	11	−8	6	3.67
VP > VA	Rt. Middle Frontal Gyrus	32	33	42	3.98

VA: Affective Violence. VP: Predatory Violence. NV: Non-violence. HC: Healthy Control.

**Table 3:** The Montreal Neurological Institute (MNI) coordinates of regions with significantly different AD, RD, and MD values in WM tissues between VP and HC groups.

	Region	MNI Coordinate			Z Score of Peak-Level Difference
		X	Y	Z	
VP > HC (AD)	Rt. Rectus	−6	23	−22	4.15
VP > HC (RD)	Rt. Rectus	−6	23	−22	4.38
VP > HC (MD)	Rt. Rectus	−6	23	−22	4.30

VA: Affective Violence. VP: Predatory Violence. NV: Non-violence. HC: Healthy Control. AD: Axial Diffusivity. RD: Radial Diffusivity. MD: Mean Diffusivity.

## Data Availability

Not applicable.
